# Transfusion

**Published:** 1878-12

**Authors:** 


TRANSFUSION.—At the meeting of the Societe Biologie, Dr. Brown-Sequard gave an interesting account of his experiments on transfusion. He had made use of different sorts of liquid for transfusion, such as normal blood, blood without its fibrine, and milk. In such case he found the results to be the same, but in the case of the milk the quantity that it was necessary to inject was more considerable than in the others. Ninety-five grammes of blood were drawn from a dog, and were replaced by the same amount of milk. Shortly after the operation (about fortyfive minutes) there was no trace of milk globules to be found in the blood, and the dog has continued in excellent health ever since the operation, which took place more than five months ago. M. Malassez found, upon examining the blood after the transfusion, a greater number of white globules than normal. In concluding his remarks, Dr. Brown-Sequard expressed the opinion that the liquid injected should be at least of a temperature of 10° to 12° C. It was preferable, he thought, to choose the arteries rather than the veins, and recommended the operation to be done very slowly, in order to allow the liquid injection to acquire the temperature of the blood. Transfusion also succeeded in animals when the blood made use of comes from a species of animals different from that of the one under experiment. It appears that Dr. Thomas, of New York, has tried the transfusion of milk on the living subject, and is convinced that it acts as well as blood.—*The Lancet.*

